# TRPM3 Is Expressed in Afferent Bladder Neurons and Is Upregulated during Bladder Inflammation

**DOI:** 10.3390/ijms23010107

**Published:** 2021-12-22

**Authors:** Matthias Vanneste, Marie Mulier, Ana Cristina Nogueira Freitas, Nele Van Ranst, Axelle Kerstens, Thomas Voets, Wouter Everaerts

**Affiliations:** 1 Laboratory of Ion Channel Research (LICR), VIB-KU Leuven Center for Brain & Disease Research, Belgium & Department of Cellular and Molecular Medicine, KU Leuven, 3000 Leuven, Belgium; matthias.vanneste@kuleuven.be (M.V.); marie.mulier@kuleuven.be (M.M.); ana.freitas@kuleuven.be (A.C.N.F.); nele.vanranst@kuleuven.be (N.V.R.); 2 VIB Bio Imaging Core, VIB-KU Leuven Center for Brain & Disease Research, Belgium & Research Group Molecular Neurobiology, Department of Neuroscience, KU Leuven, 3000 Leuven, Belgium; axelle.kerstens@kuleuven.be; 3 Laboratory of Organ Systems, Department of Development and Regeneration, KU Leuven, Belgium & Department of Urology, University Hospitals Leuven, 3000 Leuven, Belgium; wouter.everaerts@kuleuven.be

**Keywords:** TRPM3, urinary bladder, bladder inflammation, bladder pain syndrome

## Abstract

The cation channel TRPM3 is activated by heat and the neurosteroid pregnenolone sulfate. TRPM3 is expressed on sensory neurons innervating the skin, where together with TRPV1 and TRPA1, it functions as one of three redundant sensors of acute heat. Moreover, functional upregulation of TRPM3 during inflammation contributes to heat hyperalgesia. The role of TRPM3 in sensory neurons innervating internal organs such as the bladder is currently unclear. Here, using retrograde labeling and single-molecule fluorescent RNA in situ hybridization, we demonstrate expression of mRNA encoding TRPM3 in a large subset of dorsal root ganglion (DRG) neurons innervating the mouse bladder, and confirm TRPM3 channel functionality in these neurons using Fura-2-based calcium imaging. After induction of cystitis by injection of cyclophosphamide, we observed a robust increase of the functional responses to agonists of TRPM3, TRPV1, and TRPA1 in bladder-innervating DRG neurons. Cystometry and voided spot analysis in control and cyclophosphamide-treated animals did not reveal differences between wild type and TRPM3-deficient mice, indicating that TRPM3 is not critical for normal voiding. We conclude that TRPM3 is functionally expressed in a large proportion of sensory bladder afferent, but its role in bladder sensation remains to be established.

## 1. Introduction

The urinary bladder enables us to store urine effortlessly, until it reaches a certain filling state, and we perceive an urge to void. Under pathological conditions however, patients can experience pain or uncontrollable urgency even at low levels of bladder filling. Functional bladder disorders, like overactive bladder (OAB) and interstitial cystitis/bladder pain syndrome (IC/BPS) affect up to 10% of the Western population and negatively impact the quality of life of patients while imposing high socioeconomical costs on society [1,2]. Since currently available pharmacotherapies fail to effectively relief lower urinary tract symptoms in a high proportion of patients, there is a need to better understand the molecular mechanisms that underlie neuronal control of the bladder [3].

The bladder is innervated by sensory nerves that have their cell bodies in the thoracolumbar (T10-L2) and lumbosacral (L5-S1) dorsal root ganglia (DRG). These spinal afferents have specialized peripheral nerve endings within the bladder mucosa and the detrusor muscle, which express a myriad of ion channels and receptors allowing them to determine the content of the bladder. Several members of the Transient Receptor Potential (TRP) channel superfamily play pivotal roles as primary molecular sensors in the peripheral nerve ending and urothelial cells in the bladder, translating intravesical stimuli into neuronal activity and affecting voiding behavior [3]. These TRP channels, like TRPV1, TRPV4, and TRPA1, contribute to normal voiding behavior and/or bladder hypersensitivity in experimental models of cystitis [4,5,6].

Recently, TRPM3 has emerged as an important mediator of acute and inflammatory pain. TRPM3 is expressed in sensory afferents innervating the skin and activation of TRPM3 by noxious heat or by agonists such as pregnenolone sulphate (PS) or CIM0216 results in acute pain responses [7]. Together with TRPA1 and TRPV1, TRPM3 is essential for the detection of painful heat, since combined genetic deletion of these three TRP channels leads to a complete loss of noxious heat perception in mice [8]. Moreover, TRPM3 expression is upregulated during paw inflammation, resulting in inflammatory heat hyperalgesia [9,10]. Accordingly, genetic ablation or pharmacological inhibition of TRPM3 reduces heat hypersensitivity in inflammatory and neuropathic pain models, suggesting that compounds targeting TRPM3 may be developed as novel analgesic drugs [9,11,12].

In addition to the skin, nerve endings of DRG neurons also have endings in internal organs such as the bladder. It is currently unknown whether TRPM3 is expressed in these interoceptive neurons, and whether the channel contributes to (patho)physiological processes in internal organs. In this manuscript, we combined retrograde labeling with single-molecule fluorescent RNA in situ hybridization and calcium imaging to demonstrate functional expression of TRPM3 in a large proportion of DRG neurons innervating the bladder. In vivo measurements in wild type and TRPM3-deficient mice did not reveal a crucial role for TRPM3 in bladder filling/voiding, both in control conditions and in a model of cyclophosphamide (CYP)-induced cystitis. A potential role for TRPM3 in bladder sensation and pain is discussed.

## 2. Results

### 2.1. TRPM3 Is Expressed in Sensory Neurons Innervating the Bladder

**Figure 1:**
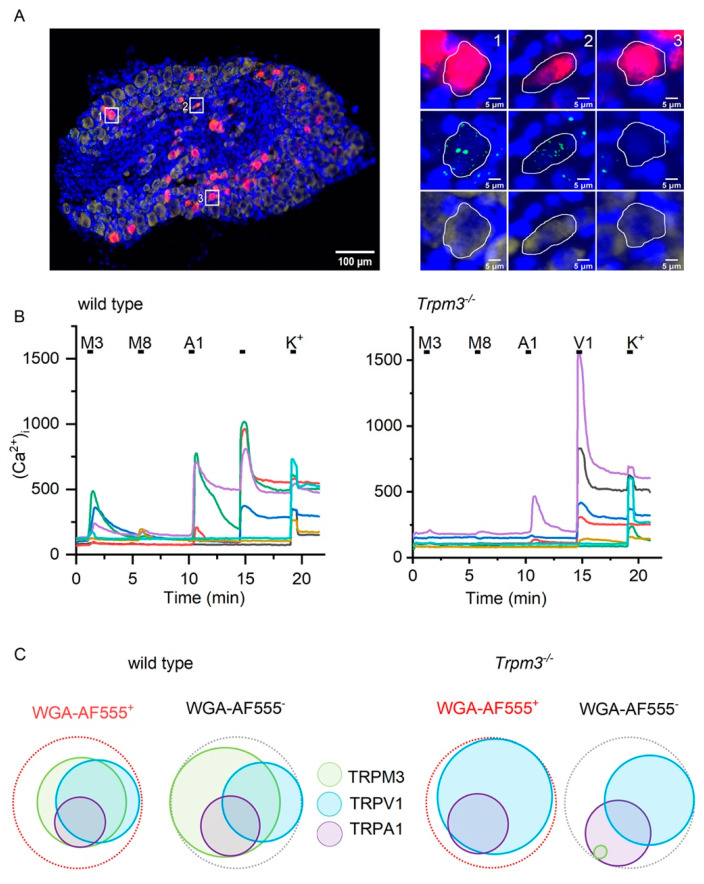
TRPM3 is expressed on bladder afferent neurons. (**A**) RNA in situ hybridization in retrogradely labeled dorsal root ganglion (DRG). Left: DRG stained with WGA-Alexafluor-647 (red), DAPI (blue), *Trpm3* (green), and *Pgp9.5* (yellow). Scale bar 100 µm. Right: High magnification images of retrogradely labeled neurons (red) expressing *Trpm3* (green) and *PgP9.5.* (**B**) Example traces of wild type (left) and *Trpm3^−/−^* (right) DRG neurons during calcium imaging. Application of agonists for TRPM3 (50 µM pregnenolone sulfate + 2 µM CIM-0216), TRPM8 (100 µM menthol), TRPA1 100 µM (mustard oil), TRPV1 (1 µM capsaicin), and finally a high-potassium solution is indicated above the trace. (**C**) Venn diagrams representing the proportions of wild type and *Trpm3^−/−^* neurons responding to application of agonists for TRPM3 (PS/CIM), TRPV1 (capsaicin), and TRPA1 (mustard oil). Bladder neurons were retrogradely labeled with WGA-AF555. Wild type: *n* = 2297 neurons from 3 mice, *Trpm3^−/−^* 1625 neurons from 3 mice.

Single-molecule fluorescent RNA in situ hybridization (RNAscope) using a *Trpm3*-selective probe was performed on thin slices of dorsal root ganglia at spinal levels L5, L6 and S1. Since these ganglia also contain cell bodies of sensory neurons projecting to other organs, including the bowel and the skin, we injected the fluorescent label WGA-AF647 into the bladder wall 7 days prior to DRG isolation, thus allowing specific identification of bladder-innervating neurons. On average, 8% of DRG neurons contained the retrograde label. In 69% of the labeled neurons, we detected the expression of *Trpm3* (Figure 1A).

Next, we performed intracellular Ca^2+^ imaging on DRG neurons from spinal levels L5, L6, and S1 isolated from animals injected with WGA-AF555. We found that 9% of all tested neurons (216/2297) were positive for WGA-AF555, and within this subset, 46% (100/216) responded to the combined application of the TRPM3 agonists pregnenolone sulfate (PS; 50 µM) and CIM0216 (2 µM) (Figure 1B,C). Responses to agonists for TRPV1 (capsaicin, 1 µM), TRPA1 (mustard oil, 100 µM) and TRPM8 (menthol, 100 µM) were detected in 40% (81/216), 15% (32/216), and 6% (12/216) of WGA-AF555^+^ neurons, respectively. Overall, these response profiles were comparable to those observed in WGA-AF555-negative neurons (Figure 1B,C).

In DRG neurons isolated from *Trpm3^−/−^* mice (Figure 1B,C), no (0/96) WGA-AF555^+^ neurons showed a detectable response to PS/CIM0216, whereas responses to the agonists for TRPV1, TRPA1, and TRPM8 were preserved (Figure 1B,C). Taken together, these data demonstrate the expression of functional TRPM3 in a large subset of DRG neurons innervating the bladder.

### 2.2. TRPM3 in Normal Bladder Function

**Figure 2:**
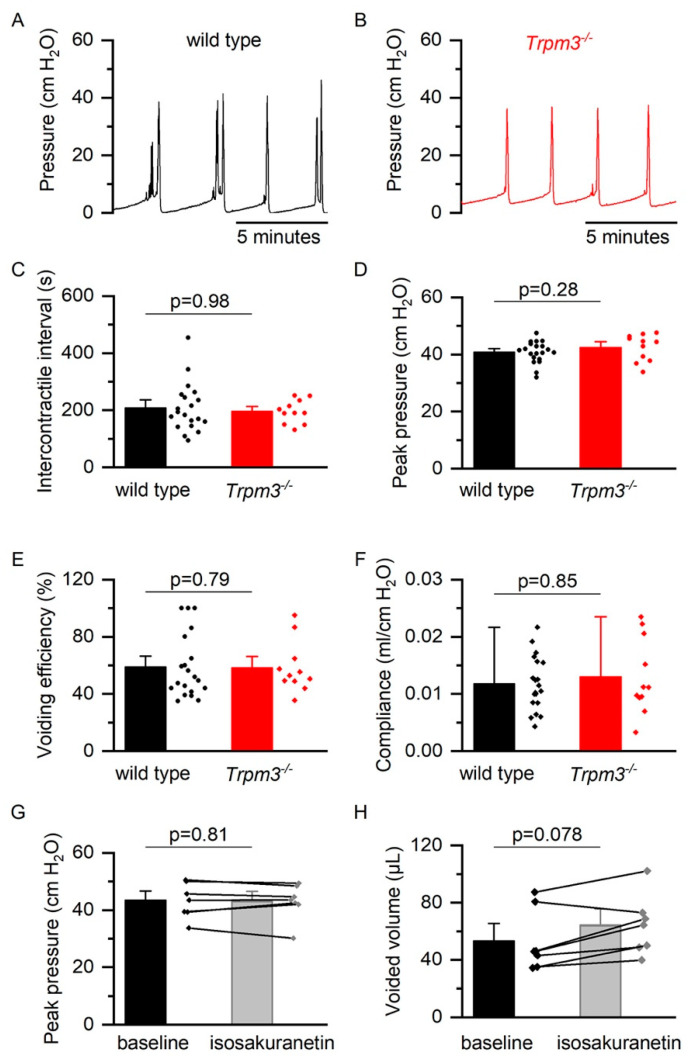
Cystometry in wild-type and *Trpm3^−/−^* mice. (**A**,**B**) Representative examples of cystometry in a wild type (*n* = 20) and *Trpm3^−/−^* (*n* = 11) mouse during bladder filling at 0.02 mL/min. (**C**–**F**) Mean (±SE) intercontractile interval, peak pressure during voiding, voiding efficiency, and bladder compliance during cystometry in wild-type and *Trpm3^−/−^* mice. (**G**,**H**) Peak pressure and voided volume during cystometry before and after intravesical isosakuranetin (10 mg/kg) administration in wild type mice.

To investigate whether TRPM3 contributes to bladder function, we performed cystometry in 10–14 weeks old female wild-type and *Trpm3^−/−^* mice. TRPM3-deficient mice displayed normal cycles of bladder filling and voiding (Figure 2B), similar to wild-type mice (Figure 2A). Mean intercontractile interval, peak pressure, voiding efficiency, and bladder compliance were not significantly different between the two groups, indicating that TRPM3 is not involved in normal bladder function (Supplementary Table S1, Figure 2C–F). Similarly, acute pharmacological inhibition of TRPM3 using intraperitoneal administration of the TRPM3 antagonist isosakuranetin, at doses of known to cause pronounced TRPM3 inhibition in vivo (2–10 mg/kg) [11], did not significantly alter intercontractile interval, voided volume, or intravesical pressures during cystometry in wild-type mice (Figure 2G,H).

**Figure 3:**
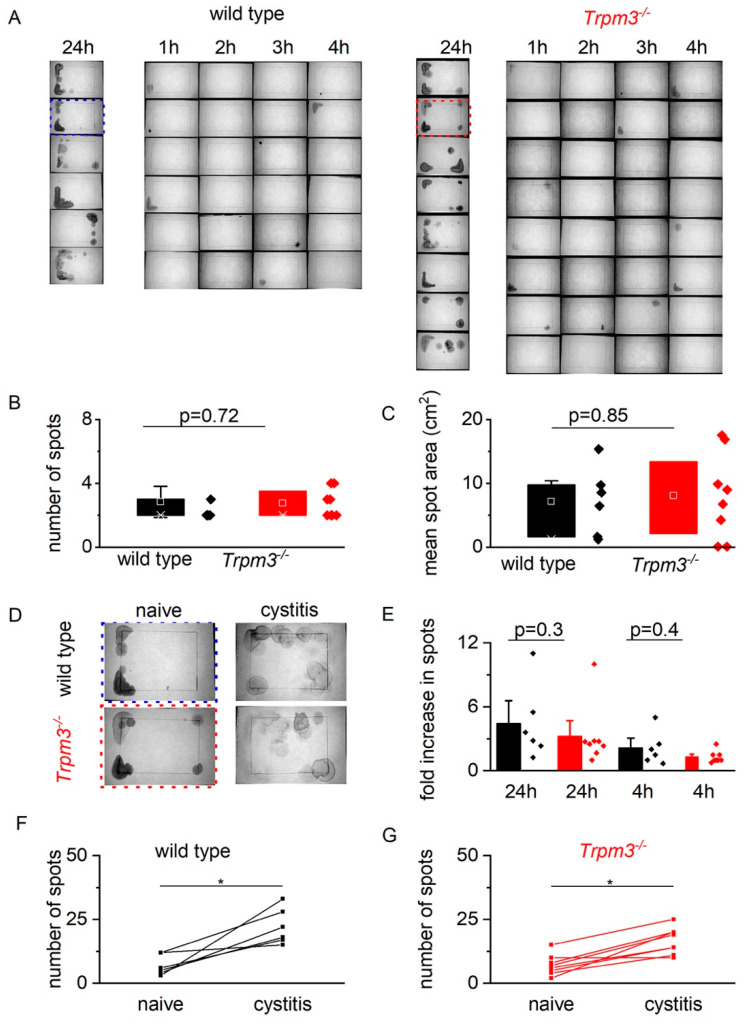
Urinary spot analysis in wild-type and *Trpm3^−/−^* mice. (**A**) Example images of voided spots in wild type (left) and *Trpm3^−/−^* mice. First column per pane: voided spots after 24 h of habituation. Next columns: voided spots per hour for four consecutive hours. (**B**,**C**) Number of voided spots and mean area per spot during 4-h spotting period in wild type (*n* = 6, black) and *Trpm3^−/−^* (*n* = 8, red) mice. (**D**) Example images of voided spots in wild type and *Trpm3^−/−^* mice before (left column) and after (right column) cyclophosphamide injection (300 mg/kg). (**E**) Fold increase (mean ± SE) in the number of voided spots per 24 h and 4 h in wild type (*n* = 6, black) and *Trpm3^−/−^* (*n* = 8, red) mice after injection with cyclophosphamide. (**F**,**G**) Number of voided spots during 24-h period in wild type (black) and *Trpm3^−/−^* (red) mice before and after cyclophosphamide (300 mg/kg) injection. * *p* < 0.05.

Next, we studied voiding behavior in awake, freely moving, and spontaneously voiding mice by performing urinary spotting experiments in male 10–14 weeks old wild-type and *Trpm3^−/−^* mice. In accordance with the cystometric recordings, the number of voided spots and average area per spot during a 4-h and 24-h period were similar in both groups, confirming a normal voiding behavior in *Trpm3^−/−^* animals (Figure 3A–C).

**Figure 4:**
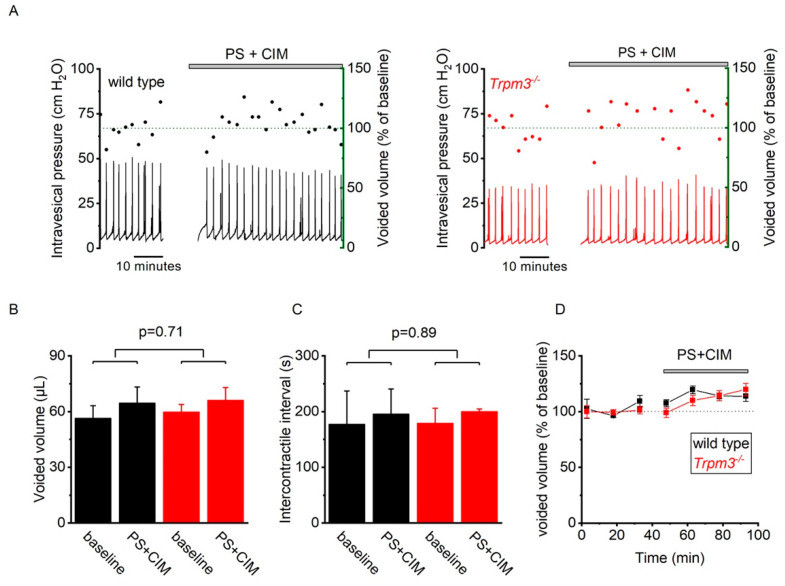
Intravesical PS + CIM instillation during cystometry. (**A**) Representative examples of intravesical pressure (black line) and voided volumes (dots) in wild type (left) and *Trpm3^−/−^* (right) mice at baseline and during intravesical instillation of PS (500 µM) and CIM-0216 (100 µM). (**B**,**C**) Mean (±SE) voided volume and intercontractile interval in wild type (black, *n* = 6) and *Trpm3^−/−^* (red, *n* = 6) mice at baseline and during intravesical instillation of PS (500 µM) and CIM-0216 (100 µM). Data were compared using two-way repeated measures ANOVA with post-hoc Tukey test. (**D**) Normalized voided volume in wild type (black) and *Trpm3^−/−^* (red) mice before and during intravesical instillation of PS (500 µM) and CIM-0216 (100 µM). Voided volume is expressed as a percentage of the average baseline voided volume.

Finally, we tested if TRPM3 on sensory nerve fibers can be activated by intravesical application of TRPM3 agonists. In these experiments, after performing baseline cystometry recordings with normal saline, the bladder of wild type and TRPM3-deficient mice was infused with a solution containing saturating concentrations of PS (500 µM) and CIM0216 (100 µM). Infusion with the TRPM3 agonists did not evoke any TRPM3-dependent changes in voiding pattern (Figure 4).

### 2.3. TRP Channel Function Is Upregulated during Bladder Inflammation

**Figure 5:**
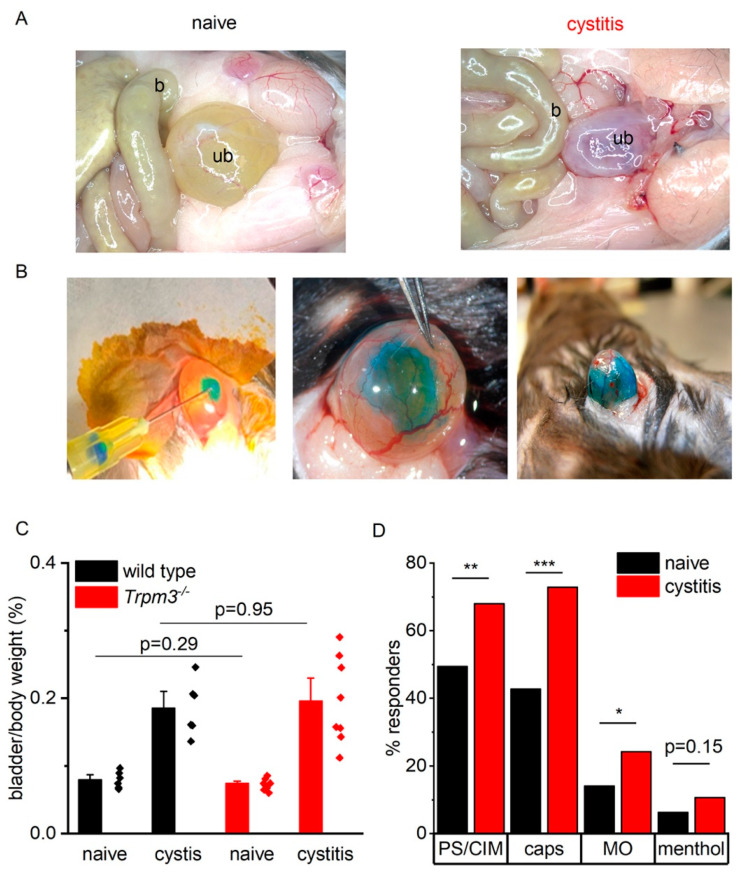
Calcium imaging during bladder inflammation. (**A**) Macroscopic view of a sham-treated bladder (left) and a CYP-treated bladder (right) 24 h after injection. b = bowel, ub = urinary bladder. (**B**) Injection of WGA-AlexaFluor 555 into the bladder wall and spreading of the dye over the bladder wall. (**C**) Bladder weight to body weight ratios in naive and CYP-treated wild type (*n* = 12) and *Trpm3^−/−^* mice (*n* = 15). (**D**) Percentage of bladder afferent neurons responding to TRP channel agonists in mice with and without bladder inflammation (*n* = 214 neurons from 3 mice and 103 neurons from 3 mice, respectively). * *p* < 0.05; ** *p* < 0.01; *** *p* < 0.001.

To detect changes in TRPM3 channel activity during bladder inflammation, we performed calcium imaging on retrogradely labeled DRG neurons isolated from control mice and mice with CYP-induced cystitis. Twenty-four hours after cyclophosphamide injection, the urinary bladder showed macroscopic signs of inflammation typified by redness, hemorrhagic spots, and edema of the bladder wall, with increased bladder weight over body weight ratios. These CYP-induced alterations were similar in wild type and *Trpm3^−/−^* mice (Figure 5A,C).

In mice with CYP-induced cystitis, the proportion of WGA-AF555-labeled neurons responding to TRPM3, TRPV1, and TRPA1 agonists was significantly higher than in sham treated animals (*p* = 0.002, *p* < 10^−6^, and *p* = 0.02, respectively). These findings indicate a functional upregulation of TRPM3, as well as TRPV1 and possibly TRPA1 during bladder inflammation (Figure 5D).

### 2.4. TRPM3 Does Not Contribute to Detrusor Hyperreflexia in Mice with CYP-Induced Cystitis

**Figure 6:**
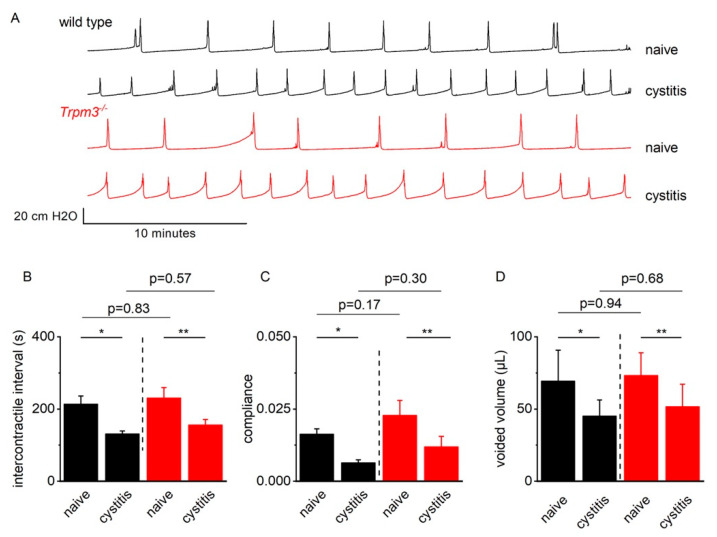
Cystometry in wild-type and *Trpm3^−/−^* mice with CYP-induced cystitis. (**A**) Representative examples of cystometry in naive and cyclophosphamide (CYP) pretreated wild type (black) and *Trpm3^−/−^* (red) mice. (**B**–**D**) Mean (±SE) intercontractile interval, bladder compliance, and voided volume during cystometry in naïve wild type (*n* = 8, black) and *Trpm3^−/−^* mice (*n* = 7, red) and CYP-pretreated wild type (*n* = 7, black) and *Trpm3^−/−^* (*n* = 8, red) mice. * *p* < 0.05; ** *p* < 0.01.

To assess a potential impact of functional TRPM3 upregulation during bladder inflammation, we compared female wild-type and *Trpm3^−/−^* mice twenty-four hours after injection of CYP. Cystometric recordings in both genotypes demonstrated a cystitis phenotype, characterized by smaller voided volumes, shorter intercontractile intervals, and lower bladder compliance, with no significant differences between wild-type and *Trpm3^−/−^* mice (Supplementary Table S2, Figure 6).

Similarly, spotting experiments in male wild-type and *Trpm3^−/−^* mice before and after injection of cyclophosphamide demonstrated a significant increase in the total number of voided spots during a 24-h period (Figure 3D,F). The observed effects of cyclophosphamide on voiding behavior were not significantly different between *Trpm3^−/−^* and wild-type mice (Figure 3D–G). Together with the observations made during cystometry, these observations argue against a role for TRPM3 in the induction of voiding symptoms during bladder inflammation in mice.

## 3. Discussion

Recent research has highlighted the role of the cation channel TRPM3 in somatosensory neurons. It is one of three sensors for acute noxious heat in the skin [8], and its activation by heat or chemical ligands such as PS of CIM0216 causes pain and neuropeptide release [7,9]. TRPM3 expression is upregulated in DRG neurons innervating inflamed skin, contributing to inflammatory hyperalgesia [9,10,11,12]. Importantly, pharmacological inhibition or genetic ablation of TRPM3 reduces hyperalgesia and ongoing pain in rodent models of inflammatory and neuropathic pain, suggesting that TRPM3 antagonists may be developed as a novel class of analgesic drugs [9,13,14,15]. However, little is known about the expression of TRPM3 in DRG neurons innervating internal organs such as the bladder, and potential (patho)physiological roles in that context remain elusive.

Here we show that TRPM3 is robustly expressed in sensory neurons that innervate the bladder. The cell bodies of bladder afferent neurons reside within the DRG at spinal levels L5, L6, and S1 in mice but constitute only a small proportion of all cells within the DRG. To discriminate between sensory neurons innervating the bladder and neurons innervating the skin, bowel, or other internal organs, we retrogradely labeled bladder afferents. Around 9% of the isolated DRG neurons were positive for WGA-AF555. Of these labeled neurons, 46% showed functional TRPM3 expression, with a large proportion of neurons co-expressing TRPV1 and to a lesser extent TRPA1. TRPM8 expression was detected in a small subset of bladder DRG neurons (8%). Our findings correspond with earlier studies showing expression of TRPV1 [16], TRPA1 [17], and TRPM8 [18] on bladder afferent neurons. 

Earlier work provided evidence for the role of sensory TRP channels in bladder hyperreactivity during cystitis (TRPV1 and TRPA1) [19,20], the detection of noxious stimuli in the bladder (TRPA1) [17], and cold-induced bladder reflexes (TRPM8) [21]. To our knowledge, however, the role of TRPM3 in bladder (patho)physiology had not been reported before. Here, we used cystometry and spotting experiments to characterize the bladder function of TRPM3-deficient mice in anesthetized and awake conditions. These experiments did not reveal any significant differences between wild type and *Trpm3^−/−^* mice. Mice lacking functional expression of *Trpm3* showed a normal voiding pattern with regular filling/voiding cycles, unaltered bladder contractility, and a normal bladder capacity. Since constitutive deletion of TRPM3 may potentially result in compensatory mechanisms obscuring a phenotype, we also assessed the effect of acute pharmacological TRPM3 inhibition on bladder function. Intraperitoneal injection of the potent TRPM3 blocker isosakuranetin, at doses that cause robust reversal of TRPM3-dependent heat hyperalgesia in inflammatory and neuropathic pain models [11], did not alter bladder function in wild-type mice. Taken together, these data indicate that TRPM3 is not essential for normal bladder function. We also did not observe any TRPM3-medicated effects on bladder function in response to intravesical instillation of the TRPM3 agonists PS and CIM0216. However, we cannot exclude that the lack of response may be due to poor penetration of these compounds through the tight barrier of the urothelium.

In line with previous findings in the skin [10], we found that functional responses to TRPM3 agonists were increased in DRG neurons that innervated an inflamed bladder. Notably, whereas TRPM3 deletion fully abrogates inflammatory heat hyperalgesia in the skin [9,12], we found here that TRPM3-deficient mice develop cyclophosphamide cystitis-induced voiding dysfunction of a similar severity as wild-type animals. These results indicate that upregulation of TRPM3 is not a key driver of bladder overactivity in this model, and that other channels such as TRPV1 and TRPA1 may contribute to the cystitis phenotype. Of note, due to the severity of bladder inflammation in the CYP-induced cystitis model, our conclusions might not be readily extrapolatable to other, less severe models of bladder inflammation such as LPS-induced [20] or auto-immune induced cystitis [22]. Finally, TRPM3 may be involved in other aspects of bladder sensation such as bladder pain, which was not directly addressed in the current study.

## 4. Materials and Methods

### 4.1. Animals

Female and male C57/Bl6J mice (Janvier Labs (France)) and *Trpm3^−/−^* mice on a C57BL/6J background [9] aged 10–14 weeks were used. Mice were housed in a conventional facility at 21 °C on a 12-h light–dark cycle with unrestricted access to food and water. All experiments were reviewed and approved by the Animal Ethical Committee of KU Leuven University.

### 4.2. Retrograde Labeling of Bladder Neurons

Bladder-specific afferent neurons were retrogradely labeled by injection of 10 µL Wheat Germ Agglutinin-conjugated (WGA) Alexa Fluor 555 or WGA-Alexa Fluor 647 (Thermo Fisher Scientific Invitrogen, Eugene, OR, USA; 0.8% in sterile PBS) as previously described [10,23,24]. Briefly, mice were anesthetized with isoflurane and the bladder was exposed through a midline incision. Using a Hamilton syringe, 10 µL label was injected into the left and right lateral bladder wall. After injection, the label spread from the injection sites, creating two expanding blebs that covered most of the entire bladder wall (Figure 5B).

After injection the syringe was left in place for 30 s to prevent leakage from the injection site. After withdrawal of the needle, a paper tissue was applied to the injection site to absorb minor leakage. After injection, the abdominal cavity was rinsed with NaCl 0.9% to wash out any leaked label and the abdomen and skin were closed. Carprofen (5 mg/kg) (Zoetis Pharma, Brussels, Belgium) was administered subcutaneously to ensure postoperative analgesia. DRG ganglia were harvested for experiments 7 days after injection of the label, as previously described [10,23,24].

### 4.3. Cyclophosphamide-Induced Cystitis

Bladder inflammation in mice is induced by intraperitoneal injection of 300 mg/kg cyclophosphamide (Sigma-Aldrich, Darmstadt, Germany) 24 h prior to tissue isolation or behavioral experiments. Cyclophosphamide is metabolized into acrolein, which is excreted through the kidneys and causes urothelial damage and bladder inflammation [25]. Control animals were injected with equal volumes of NaCl 0.9%.

### 4.4. RNA Scope

Seven days after injection of WGA-AlexaFluor 647, mice were sacrificed using CO_2_ inhalation. The dorsal root ganglia at the levels L5, L6, and S1 were collected, immersed in 10% neutral buffered formalin for 24 h, and changed to a sucrose solution. The DRGs were snap frozen in liquid nitrogen and 10 µm cryosections were cut. RNA transcripts for *Trpm3* and *Pgp9.5* were detected using the RNAscope 2.0 assay following the manufacturer’s instructions (Advanced Cell Diagnostics, Hayward, CA, United States). Probes for *mTrpm3* (cat number: 459911), *mTrpv1* (cat number: 313331), *mTrpa1* (cat number: 400211), *mTrpm8* (cat number: 420451), and *Pgp9.5* (cat number: 561861-C2) were purchased from Advanced Cell Diagnostics. The staining was performed using the RNAscope Fluorescent Multiplex Reagent Kit (cat number: 320850). Cells were stained with DAPI and mounted on the slide with Gold Antifade Mountant.

For image acquisition, a Märzhäuser Wetzlar Slide Express 2 attached to a Nikon NiE inverted microscope was used in combination with a 20× Plan Apo lambda objective lens (NA 0.75). The setup was controlled by NIS-Elements (5.21.03, Nikon Instruments Europe B.V.). For quantification of the positive cells, consecutive stainings were imaged in consecutive runs, and the images were subsequently registered using the ec-CLEM plugin in ICY (version 2.2.1.0, Institute Pasteur).

For image analysis, NIS-Elements (5.21.00, Nikon Instruments Europe B.V.) was used. To determine the percentage of labeled neurons that express *Trpm3*, the number of DRG neurons containing the WGA-AF647 label was counted (using a threshold). The contrast in the green *Trpm3* channel was enhanced, and the green signal was segmented with a bright spot detection. The number of WGA-AF647 labeled cells that contain *Trpm3* was then counted. 

### 4.5. Calcium Imaging of Bladder Afferent Neurons

Seven days after injection of WGA-AlexaFluor 555, mice were euthanized using CO_2_ and DRGs at the spinal levels L5, L6, and S1 were removed, washed in neurobasal A medium (Invitrogen, Gent, Belgium) supplemented with 10% fetal calf serum (basal medium), and then incubated for 45 min at 37 °C in a mix of 1 mg/mL collagenase and 2.5 mg/mL dispase (Gibco, Gent, Belgium). Digested ganglia were gently washed twice with basal medium and mechanically dissociated by passage through syringes fitted with increasing needle gauges. Neurons were seeded on poly-L-ornithine/laminin coated glass-bottom chambers (Fluorodish, WPI, Hertfordshire, UK) and cultured overnight at 37 °C in 5% CO_2_ in B27 (Invitrogen) supplemented neurobasal A medium, containing 2 ng/mL GDNF (Invitrogen) and 10 ng/mL NT4 (Peprotech, London, UK).

DRG neurons were loaded with 2 µM Fura-2-acetoxymethyl ester (Enzo Life Sciences, Brussels, Belgium) for 30 min at 37 °C. Fluorescence was measured during alternating illumination at 340 and 380 nm using an Eclipse Ti (Nikon) fluorescence microscopy system. During the experiment, the neurons were continuously perfused with a standard extracellular solution containing 150 mM NaCl, 6 mM KCl, 2 mM CaCl_2_, 1.5 mM MgCl_2_, 10 mM HEPES, and 10 mM glucose (pH 7.4 with NaOH) at room temperature. TRP channel agonists were applied for 30 s in the following concentrations: 50 µM pregnenolone sulfate (Sigma-Aldrich, Darmstadt, Germany), 2 µM CIM0216 (Sigma-Aldrich, Darmstadt, Germany), 1 µM capsaicin (Sigma-Aldrich, Darmstadt, Germany), 100 µM mustard oil (Sigma-Aldrich, Darmstadt, Germany), and 100 µM menthol (Sigma-Aldrich, Darmstadt, Germany). After each application, agonists were washed out for 4 min using the standard extracellular solution. At the end of every experiment, cells were perfused with a high-K^+^ solution containing 100 mM NaCl, 56 mM KCl, 2 mM CaCl_2_, 1.5 mM MgCl_2_, 10 mM HEPES, and 10 mM glucose (pH 7.4 with NaOH) to identify all excitable cells.

### 4.6. Cystometry

Cystometry was performed as previously described [26]. Mice were anesthetized using isoflurane (5% induction, 1.5% maintenance) and subcutaneous injection of urethane (1.2 mg/kg) (Sigma-Aldrich, Diegem, Belgium). During surgery, isoflurane levels were gradually lowered until urethane-only anesthesia was achieved. A PE-50 tube was implanted in the bladder dome and connected to a pressure transducer (Biopac TSD104A Pressure transducer, Biopac Systems Inc., Goleta, CA, USA) with an amplifier (Biopac DA100C, Biopac Systems Inc., Goleta, CA, USA) that is connected to the acquisition unit (Biopac MP-150, Biopac Systems Inc., Goleta, CA, USA). Through the PE-50 tube, normal saline was infused into the bladder at a constant infusion rate of 0.02 mL/min. After at least 30 min of baseline or until stable micturition cycles are observed, the measurement continued for a minimum of 20 min. Voided volumes were calculated by collecting the voided drops on filter paper and weighing them.

Isosakuranetin (Extrasynthese, Genay Cedex, France) was dissolved in DMSO and further diluted in normal saline (NaCl 0.9%). Isosakuranetin was administered via intraperitoneal injections at doses of 2 and 10 mg/kg.

Analysis of the cystometry traces was done using Acknowledge software (Acknowledge 5.0.4, Biopac Systems Inc., Goleta, CA, USA). Basal pressure is defined as the lowest recorded pressure during a single voiding cycle. Conversely, peak pressure is the highest pressure during one cycle. The pressure at which the slope of the pressure-time curve converts from a slow, gradual rise to a sudden steep increase in pressure is defined as threshold pressure [27]. Compliance is calculated during a stable filling phase as the total infused volume during this period divided by the rise in pressure. Statistical analysis was done using OriginPro (OriginPro 2018 b9.5.1.915, OriginLab Corporation) and Graphpad Prism (Prism version 9.1.1, Graphpad Software, LCC).

### 4.7. Spotting Experiments

Mice were placed in grid cages with a filter paper underneath to collect the urine. After a habituation period of 24 h, the mice remained in the cages for 4 more hours. Filter papers were changed every hour and water intake was measured. The filter papers were then photographed under UV light to visualize urine spots. Image analysis was done using ImageJ (ImageJ 1.53c, National Institutes of Health, New York, NY, USA). The number of spots, the size of individual spot, and the total spotted area were analyzed.

### 4.8. Statistical Analysis

The percentages of responding cells during calcium imaging were compared using the Chi-squared test, amplitudes of response were compared using the Mann–Whitney-U test. Cystometric and spotting parameters were compared using the Mann–Whitney-U test for comparison between two unrelated groups, Wilcoxon signed rank test for paired comparison of groups, or analysis of variance (ANOVA) with post-hoc Tukey test for comparison of more than two groups. A *p*-value < 0.05 was considered significant.

## 5. Conclusions

We demonstrated robust functional expression of the cation channel TRPM3 on sensory neurons innervating the bladder. Our findings further indicate that, both under normal circumstances and during cystitis, TRPM3 does not play a critical role in the process of bladder filling and voiding. Since antagonists of TRPM3 evoke significant analgesia in various preclinical models, the channel represents a potential novel target to treat chronic pain in patients. In that context, our present results render it unlikely that systemic inhibition of TRPM3 will have deleterious effects on bladder function, in contrast to, for instance, opioid analgesics which can lead to urinary retention. We also do not exclude that the increased functionality of TRPM3 may contribute to bladder pain associated with cystitis and that TRPM3 antagonism may cause relieve in bladder pain patients.

## Data Availability

The data analyzed and presented in this study are available from the corresponding author on request.

## Supplementary Materials

The following are available online at https://www.mdpi.com/article/10.3390/ijms23010107/s1.
