# Supplementary material for: TRPM3 Is Expressed in Afferent Bladder Neurons and Is Upregulated during Bladder Inflammation
Source: Int J Mol Sci. 2021 Dec 22;23(1):107. doi: 10.3390/ijms23010107 (PMC8745475; doi:10.3390/ijms23010107)
Supplement: Supplementary file 1 [file ijms-23-00107-s001.zip › ijms-1514210-supplementary.pdf]

Supplemantary data

Supplementary Table 1

	Wild-type	*Trpm3*$^{-/-}$	p-value
number of animals	20	11	
intercontractile interval (s)	188 (153-249)	190 (150-234)	0.98
voided volume (µl)	54 (41-72)	59 (45-76)	0.58
basal pressure (cm H_2O)	3 (2-4)	4 (2-4)	0.98
peak pressure (cm H_2O)	41 (39-43)	44 (38-46)	0.27
residual volume (µl)	55 (28-67)	45 (36-73)	0.66
voiding efficiency (%)	51 (43-72)	53 (49-65)	0.79
compliance	0.011 (0.0085-0.016)	0.011 (0.0093-0.021)	0.85
total bladder capacity (calculated)	111 (85-135)	113 (82-117)	0.69

Table S1. Parameters of cystometry in wild type and *Trpm3*$^{-/-}$ mice. Values are presented as median ± interquartile range, medians were compared using Mann-Whitney-U-test

Supplemantary Table 2

	Wild-type			*Trpm3*$^{-/-}$		
	saline	CYP	p-value	saline	CYP	p-value
number of animals	8	7		7	8	
intercontractile interval (s)	214 (43)	130 (16)	**0.00116**	230 (52)	156 (30)	**0.00379**
voided volume (µl)	69 (14)	45 (6.5)	**0.00184**	73 (14)	52 (8.3)	**0.00578**
basal pressure (cm H_2O)	2.8 (1.4)	3.9 (1.0)	**0.41678**	2.4 (1.3)	3.8 (1.1)	**0.17317**
peak pressure (cm H_2O)	42 (3.8)	35 (2.1)	**0.01413**	40 (3.8)	40 (5.6)	**0.99999**
residual volume (µl)	36 (29)	30 (30)	**0.97215**	47 (30)	8.1 (6.3)	**0.03081**
voiding efficiency (%)	70 (20)	66 (21)	**0.97922**	64 (15)	86 (9.8)	**0.07671**
compliance	0.016 (0.0035)	0.0063 (0.0019)	**0.01763**	0.023 (0.0091)	0.012 (0.0068)	**0.00824**
theoretical total bladder capacity	105 (32)	75 (31)	**0.26214**	120 (40)	60 (5.2)	**0.0029**

Table S2. Parameters of cystometry in saline and cyclophosphamide pretreated wild type and *Trpm3*$^{-/-}$ mice. Values are presented as mean ± standard deviation, differences within the groups were compared using two-way analysis of variance with post-hoc Tukey test
